# Facing the cancer-related life threat as adolescent and young adult (AYA) after initial diagnosis: A qualitative interview study

**DOI:** 10.1007/s00520-025-09466-x

**Published:** 2025-04-23

**Authors:** Mara Weiß, Carolin Wilharm, Michael Hallek, Raymond Voltz, Anne Pralong, Steffen T. Simon, Armin Tuchscherer

**Affiliations:** 1 Department of Palliative Medicine, Faculty of Medicine and University Hospital, University of Cologne, Kerpener Street 62, 50937 Cologne, Germany; 2 Department I of Internal Medicine, Faculty of Medicine and University Hospital, University of Cologne, Cologne, Germany; 3 Center for Integrated Oncology, Faculty of Medicine and University Hospital, University of Cologne, Cologne, Germany; 4 Department of Medical Oncology, Evangelic Hospital Essen Mitte, Essen, Germany

**Keywords:** Adolescents and young adults (AYA), Cancer, Initial diagnosis, Life threat, Mortality, Qualitative

## Abstract

**Purpose:**

The aim of this study was to identify coping strategies, challenges, and needs regarding the life-threatening nature of cancer from the perspective of adolescents and young adults (AYAs) shortly after their initial diagnosis.

**Methods:**

We conducted a qualitative interview study in Germany with AYAs from 18 to 39 years of age, 4–12 weeks after initial diagnosis. The interviews were transcribed verbatim, and the transcripts were analyzed using Framework Analysis.

**Results:**

Eighteen patients (7 females, 11 males, 27.2 ± 5.3 years) were included. The sudden confrontation with their mortality due to a potentially life-threatening illness stood in contrast to the feeling of youthful invincibility. One of the AYAs’ biggest concerns was burdening their relatives with the disease and the associated life threat. Many AYAs wanted to support their next of kin whilst at the same time having a great need for support from them. While there were inter-individual differences in how AYAs dealt with mortality, the most important coping strategy was hope. The attitude of healthcare professionals (HCPs), age-appropriate, individualized education, and treatment progress were important factors in this.

**Conclusion:**

Even after a recent initial diagnosis, the topic of mortality can be pertinent for AYAs. Often, a unique relationship dynamic between AYAs and their relatives arises which requires further research. HCPs play a central supporting role. Helpful conversations require appropriate timing, a strong doctor-patient relationship, and a sincere, encouraging approach. Further studies, e.g., using a longitudinal or quantitative approach, are needed.

Trial registration number: DRKS00030276, 13.09.2022

## Background

Young adults face unique challenges when diagnosed with cancer. The age range for adolescents and young adults (AYAs) with cancer varies internationally [1–3]. In German studies, the AYAs included typically range from 18 to 39 years old, as patients under the age of 18 years are cared for in pediatric departments [1, 2, 4, 5]. Annually, approximately 16,500–17,000 of them are diagnosed with cancer [6, 7]. With a 5-year survival rate exceeding 85%, their prognosis is more favorable compared to older patients [6, 8]. Accordingly, survival is a central focus in their treatment [9]. In consequence, the issue of cancer-related mortality is easily overlooked, particularly in the early phase after initial diagnosis [10, 11].

Nonetheless, receiving a cancer diagnosis at a young age can pose significant challenges. Adolescence is a stage characterized by health and future orientation, yet AYA cancer patients are confronted with a potentially life-limiting disease [3, 12–14]. Many face (their own) mortality for the first time [12, 15, 16]. Due to their youth, they have often not encountered severe illness previously, and their coping strategies are still developing [15, 17, 18]. If death or a severe course of cancer is witnessed in fellows, this exacerbates the situation [12, 15].

Supporting AYA cancer patients with this issue presents a challenge for clinicians as well [11, 14, 17–22]. Clinicians feel uncomfortable raising the sensitive topic of mortality, hence conversations about the life-threatening aspect of cancer are often avoided or delayed [13, 19]. Concerns are that open communication about mortality might undermine hope and overburden patients [18, 20, 22]. There is a lack of evidence to guide practitioners in this conflict.

As the preexisting research on the life-threatening aspects of cancer predominantly focuses on AYAs with late-stage cancer, there is very limited understanding of the specific issues faced by AYAs, who have only received the diagnosis within months [10, 18, 23–26]. It remains unclear when and in what form the need to discuss mortality becomes relevant to them [10, 11].

The aim of this study therefore is to identify coping strategies, challenges, and needs regarding the life-threatening nature of the AYAs’ cancer disease shortly after initial diagnosis and to derive implications for future clinical research and practice from this in-depth insight.

## Methods

### Study design

We conducted a qualitative interview study with AYA cancer patients using a semi-structured interview guide. The study was approved by the Ethical Review Committee of the Medical Faculty of the University of Cologne (Ref. Nr. 20 - 1472_1) and is registered in the German Register of Clinical Studies (DRKS00030277).

### Participants

The study participants had to have the initial diagnosis of an oncological disease (C-diagnosis in accordance with ICD-10-GM) at an age ≥ 18 years and ≤ 39 years. They had to be diagnosed 4–12 weeks prior to taking part in the study.

In order to achieve comparability, we followed other German studies and targeted 18- to 39-year-old patients, even though some international AYA definitions involve patients from 15 years of age [2, 27].

Patients who could not be surveyed due to severe cognitive impairment or lack of German language skills were excluded.

### Patient recruitment

Recruitment was conducted at the University Hospital Cologne and the Center for Integrated Oncology (CIO) Cologne, as part of one of the largest cancer centers in Germany. A purposive sampling strategy was employed. After obtaining consent from the treating physician, patients meeting the inclusion criteria were thoroughly informed about the study by the interviewer (MW). None of them were previously known to the researcher. Recruitment continued until saturation was reached, defined as the point at which no additional findings emerged by increasing the sample size [28, 29].

### Data collection

The audio-recorded interviews were conducted in person at University Hospital Cologne between May 2021 and September 2022. They were carried out by MW, a female medical student of similar age to the participants, who received extensive training and supervision from STS, an expert with considerable experience in conducting similar studies.

Informed consent and demographic data were obtained from all participants prior to the interviews.

A semi-structured interview guide (Table 1) was used, which was developed through comprehensive literature review and in collaboration with experts of the research team, including doctors and experienced scientists specialized in palliative medicine and hematology-oncology [30]. It was refined in consultation with AYA cancer patients.

**Table 1:** Semi-structured interview guide (excerpt)

Introduction	
Introduction of the interviewer Explanation of the procedure and aim of the study Ensuring the well-being of participants	“This is a space where you have the opportunity to share any thoughts you feel are important. The aim is to understand you and your life situation.” “Before we start the interview, I would like to point out that your well-being has priority. This also means that you can and should interrupt at any time if the situation requires it.” “Do you have any remaining questions?”
General insight	
Previous encounters with dying and death Overall attitude towards death Change in attitude due to the cancer diagnosis	“To what extent have you engaged with the topic death and dying?” “Do you have experiences with death in your personal surroundings?” “Has the diagnosis of cancer affected your attitude towards mortality?”
Supportive and vulnerability factors, stressors, and needs	
Identify coping mechanisms Preferred and actual approach of personal and professional surroundings	“How do you deal with the topic of mortality?” “When it comes to your mortality, what kind of behavior do you perceive in your surroundings (doctors/fellow patients/family/friends/partner/society/...)?” “What could a supporting approach look like?” “What is more harmful than beneficial?”
Closure	
Completion	“Is there anything else you would like to add?” “Is there any aspect important to you that has not yet been addressed?”

After the interview, a debriefing was held. Field notes were taken to capture non-verbal contextual information [31].

### Data processing and analysis

The interviews were anonymized and transcribed verbatim following predefined transcription rules using MAXQDA (VERBI, 2022) [32]. The transcripts were analyzed following Framework Analysis by Ritchie and Lewis. To enhance objectivity, the interviews were double-coded by two independent researchers. Discrepant findings were critically discussed with experienced colleagues (StS, AT, and CW) until consensus was reached.

## Results

### Study population

The study was introduced to 21 patients, of whom 18 agreed to participate. Reasons for refusal were poor medical condition (1 patient), severe psychological distress (1 patient), plus one patient giving no reason. The characteristics of the study population are shown in Table 2.

**Table 2:** Patient characteristics

AYA with cancer (*n* = 18)	Measure (*n*)
Age at diagnosis	(27.2 ± 5.3 years)
18–28 years	11
29–39 years	7
Gender	
Male	11
Female	7
Diagnosis	
Hodgkin’s lymphoma	6
Leukemia	6
Non-Hodgkin lymphoma	4
Testicular cancer	1
Sarcoma	1
Place of care	
Inpatient	10
Outpatient	8
Education/employment status at diagnosis	
Employee	8
Student	6
Not employed/in education	2
Apprenticeship	1
Doctorate	1
Relationship status at diagnosis	
Single	10
Committed partnership	5
Married	3
Children	
No	15
Yes	3

The interviews lasted between 37 and 81 min (mean 58 min). All except one patient decided to be interviewed alone.

### The contradictions of being young and just diagnosed with cancer

Confronting the life-threatening nature of their illness presented a significant challenge for all participants. The degree to which patients were concerned with protecting their relatives or with receiving care from them varied—sometimes both attitudes coexisted. The coping with mortality also differed: while some avoided the topic entirely, others engaged with it intensively. A common topic among all interviewees was the coexistence of hope for recovery alongside a profound fear, particularly regarding the dying process.

### Sense of youthful invincibility vs. facing mortality

“There are these quotes that humans always feel immortal. That’s how it is. […] Never in my life have I thought about my death before the diagnosis.”, male, 24 y., Leukemia

“You have goals, for example, I always wanted to travel to Africa or simply travel the world or learn new skills, play an instrument or whatever. And then you think to yourself: […] Will I still be able to do all that? Will I still have the time?”, male, 25 y., Hodgkin’s Lymphoma

Prior to the diagnosis, most participants had a distant relationship with dying and death, primarily associated with old age or serious illnesses in others.

However, the cancer diagnosis abruptly shattered their sense of youthful invincibility, with key moments of realization occurring within the first weeks (e.g., receiving the diagnosis, beginning treatment, first hospitalization, experiencing adverse effects, and witnessing death in peers). Facing a potentially life-limiting disease suddenly confronted them with their own mortality, making death more tangible and threatening. The AYAs became aware that their life trajectory could take a very unexpected turn, undermining the certainty that they still had their whole lives ahead of them. This uncertainty was compounded by the absence of any absolute guarantee of treatment success.

In this phase, which is fundamental to the entire course of life, some patients worried that they may not have enough time to achieve their life goals.

### Supporting relatives vs. being supported by them

“That is the feeling I get a lot with my parents […]: I'm the happiest and somehow the most positive one here. They’re almost worse off than me. And then to confront them with a topic like that [...] I don’t think it has ‘fit’ so far.”, female, 26 y., Non-Hodgkin’s Lymphoma

“I just know that the whole subject [mortality] scares my partner incredibly much. […] And that’s why I tended to hold back myself.”, female, 26 y., Non-Hodgkin’s Lymphoma

Most AYAs described a sense of responsibility for the well-being of their next of kin. Many perceived that their family and friends suffered from a strong but mostly unspoken fear that they might die from their illness. In an effort to shield their loved ones from this distress, they attempted to adopt a particularly unconcerned and positive façade. This frequently involved deliberately avoiding the topic of mortality and death.

“When you lie alone in bed at night and think: 'Oh God, am I going to be healthy again?’, it’s the family that helps you to stay optimistic.”, female, 19 y., Hodgkin’s Lymphoma

Simultaneously, for most AYAs, the primary role of relatives was to offer encouragement, motivation, comfort, and hope.

“I have the feeling that death is not really discussed. […] Especially not with my parents […] and particularly not when it comes to me. Because it’s not my turn yet in terms of age.”, female, 27 y., Hodgkin’s Lymphoma

Since the topic of dying and death did not align with either attitude, it was sometimes treated as a taboo.

### Avoiding vs. actively engaging with mortality

“But I don’t think about dying. I don’t want to, because it drags me down. And then, I don’t even need to participate in all this here. […] There’s no point in this otherwise.”, male, 29 y., Testicular Cancer

“I haven’t thought about it [death]. Because – it’s nothing to me. That little bit of cancer, I laugh at it.”, male, 23 y., Leukemia

Some interviewees found thoughts about their own mortality overwhelming, describing them as draining energy needed to endure therapy and its adverse effects. For them, the key coping strategy was to suppress thoughts of death or to trivialize the severity of the diagnosis. They preferred to avoid conversations about the life-threatening aspect of cancer unless absolutely necessary.

“The more you talk about a thing, the more it loses its horror. And especially in this context [death and dying], I imagine that it becomes easier.”, female, 27 y., Hodgkin’s Lymphoma

“I think many people are concerned that if someone talks about death, they no longer have the will to live, which of course is absolutely not the case. […] I do wish that people would handle it more openly.”, male, 24 y., Leukemia

Simultaneously, some respondents expressed a desire to normalize discussions about death and dying, even at their young age and early stage of disease. They stated that this approach reduced the horror around death, as it made mortality seem less extraordinary.

For some, this involved discussing their wishes with relatives and HCPs, allowing them to feel prepared in case of a change in goals of care. Others sought opportunities to share their thoughts and fears about death, e.g., through psycho-oncological support.

“I know that everything I’ve experienced in my life so far has been really cool. […] I don’t have the feeling that I must catch up or achieve anything. But if I were to die tomorrow, then that’s okay.”, female, 27 y., Hodgkin’s Lymphoma

Additionally, some interviewees coped with dying and death through reflection, which often prompted a deeper contemplation of life. These AYAs described their retrospective view on life as accompanied by a sense of gratitude and peace, especially when participants felt they had nothing to regret. In some cases, the prospective view led to a greater appreciation for the remaining lifetime or to a reevaluation of life goals.

### Hope for a cure vs. fear of dying

“I think: well, I’m not going to die now, but: I can do it. The cancer will get lost really quick and be defeated.”, female, 27 y., Hodgkin’s Lymphoma

The primary coping strategies for dealing with mortality were hope and determination: all interviewees established a clear goal of achieving recovery and drew strength from this conviction.

“I’m not afraid of [death] itself – I’m just afraid of the dying process.”, male, 29 y., Leukemia

Despite their hope for a cure, respondents expressed a fear of the dying process, which they associated with the fear of physical and emotional pain. Most AYAs described a fear of dying rather than of death itself, as they expected all sensations to be absent in the state of death. In some cases, death was even perceived as a form of liberation.

“I think it’s important that you are always able to decide for yourself. So, before I would peg out miserably here, I would find another way. […] I decide for myself.”, male, 23 y., Leukemia

Some participants were concerned about the loss of autonomy and control through the dying process. These patients emphasized the importance of not becoming helpless but of retaining the ability to make and enforce their own decisions, if necessary, using a proxy. For a few participants—exclusively male in this study—this concern extended to the point where they stated they would rather choose suicide than endure the dying process.

### Support from health care providers (HCPs)

HCPs play a crucial role in supporting AYAs in coping with the cancer-related life threat. Beyond maintaining an optimistic attitude, their key responsibilities include providing comprehensible and honest communication of information, fostering a respectful doctor-patient relationship, and approaching the topic of mortality with confidence.

### Fostering hope

“The doctors are optimistic [...], if you realize that as a patient, then you have more courage again. [...] If the doctors are optimistic, then I’m optimistic too.”, male, 22 y., Leukemia

Most of the participants preferred a physician’s approach that prioritized encouragement and reassurance. They valued a focus on their current situation, where an emphasis on hope was perceived as crucial. Still, they also emphasized the importance of receiving an honest assessment of their chances of recovery.

### Informed understanding

“I know exactly what’s going on, the doctors explained everything to me [...]. I know exactly what is planned, what will be done to me. That just gives me optimism.”, male, 22 y., Leukemia

A key foundation for trust and hope was a clear understanding of treatment options and therapeutic progress. To achieve comprehension, the surveyed AYAs wished for age-appropriate and individualized information.

For example, many participants considered it helpful to subdivide the therapy process into smaller steps by defining intermediate goals. They described how it prevented them from feeling overwhelmed and enhanced the value of the small successes, which could serve as pro-arguments for survival.

When physicians convey information, AYAs emphasized the importance of receiving all crucial details, and yet not being overloaded. Some patients highlighted the need for physicians to take their time, engage in repeated conversations, and provide sufficient space for their questions.

### Facilitating communication

“I remember sitting there, watching myself from the outside and thinking: ‘This should be the moment that’s really bad’. But [my doctor] spoke with such confidence, calm, and normalcy that I sat there and thought: ‘Oh well, okay. So, what do we do now?’ (laughs).”, female, 24 y., Hodgkin’s Lymphoma

To establish a trusting doctor-patient relationship that provides the opportunity to talk about mortality, many patients emphasized the importance of being met at eye level. For them, this included using adapted, understandable language, an emphatic approach, and, for some, informal address.

When speaking about death, the physician’s attitude was seen as crucial. Many patients expressed a desire for their doctor to convey a sense of sovereignty, as their attitude provided stability and orientation during this potentially overwhelming conversation.

## Discussion

To the best of our knowledge, this is the first qualitative study to explore AYAs’ coping with death and dying soon after initial diagnosis.

Our findings expand current understanding by demonstrating that even in the brief period after initial diagnosis, mortality often was an issue for the AYAs. While all participants remained determined to achieve a cure, their approaches to mortality varied markedly, with some avoiding the topic entirely, while others emphasized the value of communication and reflection. We found a similar contrast in the interaction with relatives: while some patients felt responsible for their loved ones’ well-being, other AYAs relied on them for (emotional) support.

Ultimately, we identified opportunities for HCPs to support AYAs in dealing with mortality.

Consistent with prior research, we confirm that a cancer diagnosis disrupts AYAs’ sense of youthful invincibility, forcing them to suddenly confront their finitude of existence and provoking profound uncertainty [3, 12, 13, 16]. The current evidence shows that thoughts of, as well as worries about, death and dying are not directly associated with poor prognosis in AYAs [11, 13, 22]. However, our findings refine this understanding by illustrating that fear of dying can be co-temporal with receiving the diagnosis.

While previous studies advocate for integrating end-of-life (EoL) discussions early into the routine of treatment, our results specify that AYAs may benefit from such conversations even in the initial phase of their illness [11, 18, 21, 22].

Existing literature indicates that while many AYAs value conversations about death, only a minority engage in such conversations with their loved ones [17, 20]. Our study clarifies specific barriers impeding these discussions, such as concerns about falsely expressing a loss of hope and the will to live, or about overburdening loved ones. Our study also deepens the understanding of AYAs’ role within family dynamics. Prior research acknowledges the protective role of AYAs as well as the mutual responsibility of protecting each other from emotional distress within families [3, 5, 33, 34]. We further elucidate how their transition from ongoing familial dependence to emerging autonomy may influence communication about mortality.

Moreover, we provide a more nuanced perspective on avoidance as a coping strategy. Previous studies stated “avoidance” as an important coping strategy that should be respected [11, 12, 16, 17, 22, 35]. Our findings add how, for some AYAs, it serves as an adaptive mechanism to conserve emotional resources for enduring treatment.

Consequently, it is crucial for HCPs not to initiate these challenging conversations during an acute state of crisis, and, in agreement with our findings, to maintain the concept of hope despite the seriousness of the topic [11, 14, 21]. The data indicates that a trusting doctor-patient relationship, honesty, and a willingness to discuss the potentially overwhelming topic of death with the AYA patient qualify an HCP as a suitable conversation partner; consideration should also be given to assigning non-physician or specially trained personnel to this task [14, 17, 18, 21].

Our findings emphasize that a calm and confident demeanor in physicians is essential for facilitating discussions on mortality. Competently conducted conversations about death can strengthen the confidence and even enhance hope in AYAs [11, 14, 22]. The opportunity to discuss their attitude towards dying and death can restore a sense of control and autonomy that some AYAs lose when confronted with a cancer diagnosis [13, 33].

### Limitations and strengths

While the present study, as is usual in qualitative research, cannot be generalized due to its small sample size, it is explorative to address important topics for future investigation.

Certainly, the study should be interpreted in light of its limitations. A selection bias could have occurred, as participation in the study required a general willingness to talk about death. In addition, the interviews were conducted during the COVID-19 pandemic, which may have added emotional burden.

## Conclusion

Regardless of prognosis and time since initial diagnosis, the topic of mortality can be relevant for AYAs. For many AYAs, the opportunity to talk about mortality is important, but initiating conversations remains a significant challenge. Clinicians should exercise caution, as the topic of death and dying can be overwhelming, and respect the need for repression in AYAs. Competently conducted conversations about dying and death are perceived as helpful by those AYAs who express a willingness to engage with the topic. Key conditions include appropriate, crisis-free timing, a stable doctor–patient relationship, sufficient time, and authenticity on the part of the HCP. Ideally, physicians should be prepared to address death and dying in an honest, patient-adapted, calm, and empathetic manner.

Future qualitative studies would benefit from heterogeneous cohorts regarding age, gender, cancer diagnosis, and prognosis. A longitudinal approach would also be valuable to capture the specific challenges, needs, and coping strategies related to mortality throughout the illness trajectory, from diagnosis to potential survivorship or end-of-life. Regarding prognosis, it may be valuable to investigate AYAs’ understanding of prognosis and how it was communicated by HCPs. Additionally, there is a need for further investigation into the unique relationship between AYAs and their relatives, with a focus on integrating support services for relatives into specialized care programs for AYAs. A subsequent step could involve adopting a quantitative approach, such as utilizing questionnaires targeting larger cohorts.

## Declarations

### Data availability

No datasets were generated or analysed during the current study.
